# Role of Boron in Assisting the Super-Enhancement of Emissions from Carbon-Implanted Silicon

**DOI:** 10.3390/ma16052070

**Published:** 2023-03-02

**Authors:** Nurul Ellena Abdul Razak, Chang Fu Dee, Morgan Madhuku, Ishaq Ahmad, Edward Yi Chang, Hung Wei Yu, Burhanuddin Yeop Majlis, Dilla Duryha Berhanuddin

**Affiliations:** 1 Institute of Microengineering and Nanoelectronics (IMEN), Universiti Kebangsaan Malaysia (UKM), Bangi 43600, Malaysia; 2 iThemba Laboratory for Accelerator Based Science (LABS), Johannesburg 2050, South Africa; 3 NPU-NCP Joint International Research Center on Advanced Nanomaterials and Defect Engineering, National Centre for Physics, Islamabad 44000, Pakistan; 4 Department of Materials Science and Engineering, National Yang-Ming Chiao-Tung University, Hsinchu 30010, Taiwan; 5 Department of Electronic Engineering, International College of Semiconductor Technology, National Yang-Ming Chiao-Tung University, Hsinchu 30010, Taiwan

**Keywords:** silicon, implantation, photoluminescence

## Abstract

The super enhancement of silicon band edge luminescence when co-implanted with boron and carbon is reported. The role of boron in the band edge emissions in silicon was investigated by deliberately introducing defects into the lattice structures. We aimed to increase the light emission intensity from silicon by boron implantation, leading to the formation of dislocation loops between the lattice structures. The silicon samples were doped with a high concentration of carbon before boron implantation and then annealed at a high temperature to activate the dopants into substitutional lattice sites. Photoluminescence (PL) measurements were performed to observe the emissions at the near-infrared region. The temperatures were varied from 10 K to 100 K to study the effect of temperature on the peak luminescence intensity. Two main peaks could be seen at ~1112 and 1170 nm by observing the PL spectra. The intensities shown by both peaks in the samples incorporated with boron are significantly higher than those in pristine silicon samples, and the highest intensity in the former was 600 times greater than that in the latter. Transmission electron microscopy (TEM) was used to study the structure of post-implant and post-anneal silicon sample. The dislocation loops were observed in the sample. Through a technique compatible with mature silicon processing technology, the results of this study will greatly contribute to the development of all Si-based photonic systems and quantum technologies.

## 1. Introduction

Silicon photonics is currently one of the most exciting fields in semiconductor technology. It holds the future to ultrafast data transmission within computer chips and telecommunication by integrating optical components, such as light sources, modulators photodetectors and passive components, on a single platform. The implementation of this technology for photonics devices results in low costs, high-level integration and improved reliability.

The only missing element for a monolithically integrated silicon-based photonics system is an efficient light source or laser. Silicon has an indirect bandgap, thereby lowering the probability of radiative recombination and prolonging the e–h radiative lifetime. Therefore, fast nonradiative recombination routes dominate over the slow radiative routes in silicon materials. Radiative emissions in silicon are almost entirely absent at room temperature. Nevertheless, in theory, radiative emissions can still be improved by preventing recombination via nonradiative routes.

**Figure 1:**
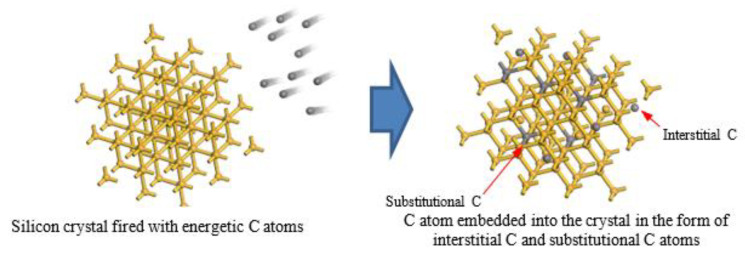
Lattice structure formation during carbon implantation. Carbon ion displaces the silicon from the lattice position to become carbon substitutional (*Cs*). The displaced silicon atom, called self-interstitial (*I*), can freely move between the lattice. Consequently, the mobile silicon (*I*) returns into the lattice site; thus, *Cs* becomes carbon interstitial (*Ci*). The interaction of *CsCi* with *I* has been proven to significantly increase the luminescence from silicon [3].

Despite the poor performance of silicon as an optical emitter due to its indirect bandgap nature, extensive effort has been given to circumvent this limitation. Techniques that have been used previously include the manipulation of point defect centers [1,2,3,4,5], Raman conversion [6,7,8,9], dislocation engineering [10,11,12,13], the incorporation of rare earth elements [14,15,16] and the epitaxial growth of III–V materials [17,18,19]. Methods such as point defect and dislocation engineering have advantages due to the utilization of tools, such as an ion implanter and rapid thermal annealer (RTA), which are among the standard processes in CMOS technology [3,20]. Ion implantation is a crucial tool, particularly in mature silicon technology, semiconductor device fabrication and advanced material research. The double implantation of carbon has been proven to assist the creation of an optically active point defect, the G-center, by producing a carbon substitutional (*Cs*) and carbon interstitial (*Ci*) pair, *CsCi*, as illustrated in Figure 1. Subsequent heat treatment by using RTA is also crucial to activating and mobilizing implanted ions to the substitutional or interstitial sites.

The dislocation engineering approach relies on the controlled introduction of extended defects, particularly dislocation loops. Loop formation requires the introduction of extra Si atoms, which can be easily achieved using ion implantation. Either excess Si atoms can be implanted directly, or the implantation of dopant species can be used. Then, excess Si interstitials are generated when the mobility of the dopant is activated, and a Si atom is displaced from a substitutional site. These Si interstitials can be aggregated into dislocation loops with a postimplant anneal. Strain is subsequently induced on the structure and increases the bandgap energy around the loop by ~0.75 eV [21]. The potential barrier prevents the excited carrier from recombining using the usual, nonradiative route. Thus, the electrons are obliged to recombine radiatively. Dislocation loops formed after the controlled implantation of carbon on silicon have also been reported [21].

We report in this paper the super-enhancement of the silicon bandgap emissions at ~1170 nm when carbon and boron are carefully introduced via ion implantation and subsequent heat treatment to activate the dopant whilst forming the necessary p–n junctions for future device fabrication. Photoluminescence (PL) measurements were performed to observe the peak intensity in samples doped with only carbon or boron and samples coimplanted with both. Silicon codoped with carbon and boron at a specific condition showed the highest emission intensity of up to 600 times greater than that in samples without carbon and boron. The composition of damage was observed, particularly the existence of dislocation loops by using high-resolution transmission electron microscopy (HRTEM). The method reported in this paper utilizes only ULSI technology-compatible processes, such as ion implantation and high-temperature annealing, which are vital for the future development of a silicon photonics integration system.

## 2. Materials and Methods

N-type silicon wafers (100) with a thickness of 525 μm and resistivity of 0.001 Ω·cm were used in this experiment. One sample was kept as the reference without any implantation. All samples were implanted using a Varian 200-20A2F ion implanter. Nine samples were implanted with single and double carbon implantation at different energy levels of 10, 20, 30 and 50 keV with a dose of 4 × 10^13^ cm^−2^. The angle of incident is 7° to minimize the effect of ion channeling [22]. The nine samples were then divided into (i) three samples without boron implantation, (ii) three samples with 10 keV of boron implantation and (iii) three samples with 30 keV of boron implantation. The remaining two samples were implanted with only 10 or 30 keV of boron. The samples were subsequently rapidly thermally annealed using rapid thermal annealer (RTA) in a nitrogen atmosphere for 20 s at 1000 °C and 60 s at 950 °C after carbon and boron implantation, respectively. Photoluminescence (PL) measurements were performed in the temperature range of 10–100 K across the 1000 nm to 1400 nm wavelength region. The samples were mounted in an Optistat DN2 cryostat, with continuous flow of helium-cooled cryostat and excited by a continuous wave 532 nm wavelength diode-pumped solid-state laser with 1.5 mm ± 0.1 mm spot size. The emitted PL was collected by Cassegrain lens and focused onto the 2 mm entrance slit of the monochromator with 600 g/mm, which was then detected by a Ge photodiode. A phase-sensitive lock-in amplifier detection technique was used for the measured PL to eliminate the background light. The importance of the variation is to study the effect of boron implantation and the temperature on the light emissions from silicon.

The details of the samples are shown in Table 1.

**Figure 2:**
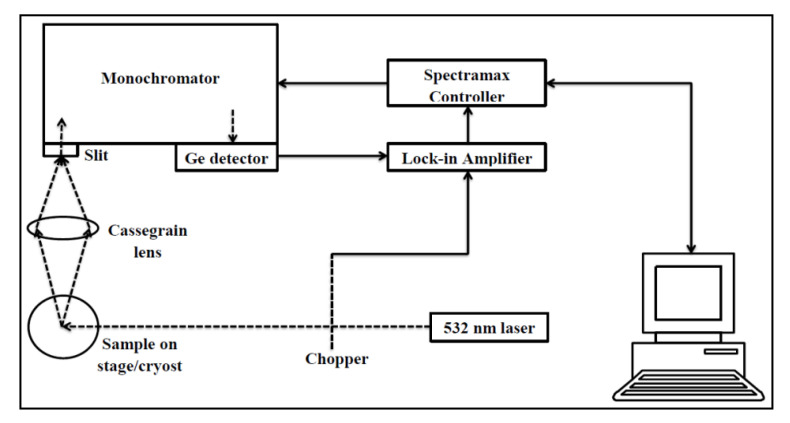
PL experimental setup. The samples were placed in the cryostat.

The experimental setup is shown in Figure 2.

## 3. Results

**Figure 3:**
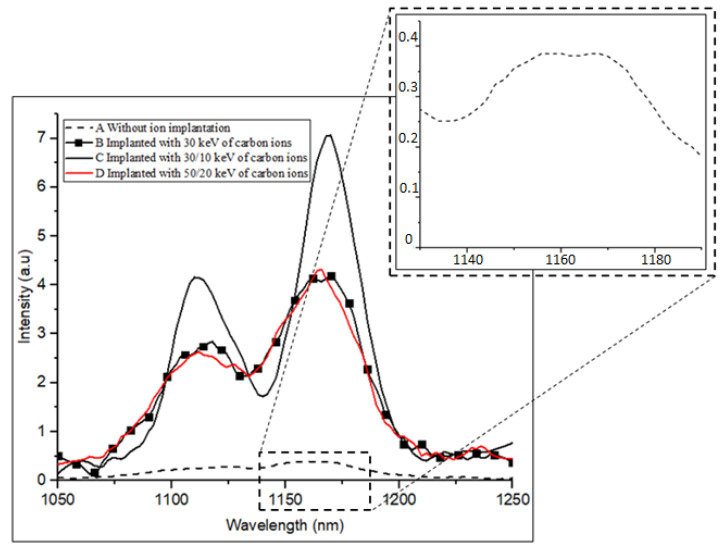
PL spectrum of samples A, B, C and D at 80 K. Sample A is the reference sample without ion implantation. Sample B is implanted with 30 keV of carbon ions, whereas samples C and D have double implantation of 30/10 and 50/20 keV of carbon ions, respectively. FWHM for the highest peak of sample C is 32 nm (29 meV). The inset shows the peak emission in sample A. The laser power was 200 mW.

**Figure 4:**
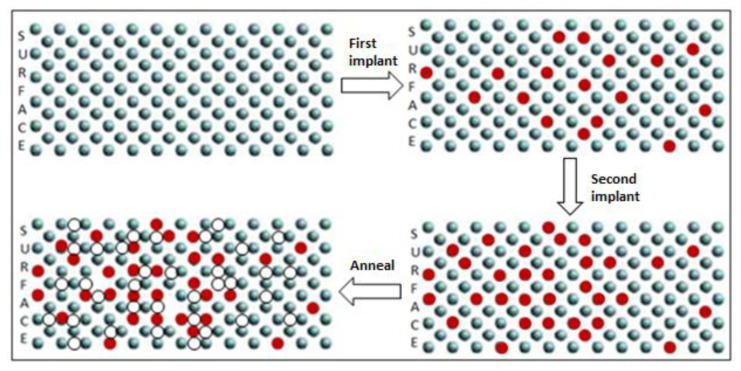
The carbon implantation during first implantation and second implantation. The silicon in crystal structure is bombarded with carbon ions. During the first implantation, carbon ions randomly fill the silicon lattice sites. The distributed substitutional carbons formed close to the surface after the second implantation are more uniform than those formed after the first implantation. Finally, rapid thermal annealing is performed to activate the dopants and create the dislocation loops. Red color is carbon ion, whereas white color is interstitial. The letters ‘SURFACE’ denotes the surface of the silicon substrate.

**Figure 5:**
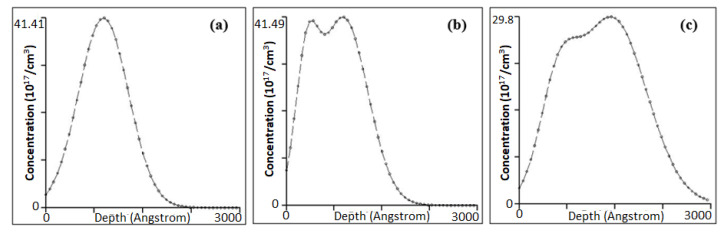
Carbon implant profile from SUSPRE simulation for (**a**) sample B, (**b**) sample C and (**c**) sample D. Sample B is implanted with 30 keV of carbon, whereas samples C and D are double implanted with 30/10 keV and 50/20 keV of carbon, respectively.

Various samples with different implant conditions were used in this study as shown in Figure 1. Figure 3 shows the PL spectrum from samples A (reference sample without implantation), B, C and D after being implanted with different energy levels of carbon at 30, 30/10 and 50/20 keV, respectively, at 80 K. The emissions at ~1170 nm of all the samples implanted with carbon are higher than those of the reference samples. The two distinct peaks are observed in all samples, excluding the reference sample. The first peak at 1112 nm is the band-to-band emission from the silicon substrate, whereas the second peak at 1170 nm is the band-to-band emission due to the heavily doped layer near the surface [23]. Sample C with the double implantation of carbon at 30 and 10 keV presents the highest peak emission with a FWHM of 32 nm (29 meV), which is attributed to the uniformity of the dislocation distribution near the Si surface, as shown in Figure 4. The lateral broadening distribution produced by double implantation in which the damage area is increased is higher than that produced by single implantation. Double implantation also forms a flat carbon profile and an improved uniformity of the carbon concentration along the depth of the samples [3,20]. The damage from implantation subsequently produces vacancies that are crucial in producing high emissions in silicon. Moreover, sample B has less uniformity than the other samples because of the single implant distribution, whereas the damage formations in sample D are located deeply in the silicon lattice, as shown in its profile distribution using SUSPRE in Figure 5. SUSPRE or the Surrey University Sputter Profile Resolution from Energy Deposition simulation is used to study the damage profile in silicon. It has been showed that its results are as accurate as those of SRIM [24]. The light absorption efficiency in Si decreases exponentially with depth. Thus, forming the optically active region spread near the surface is crucial. The silicon bandgap emissions in sample C increased by up to 120 times higher than the emissions in intrinsic silicon structure.

**Figure 6:**
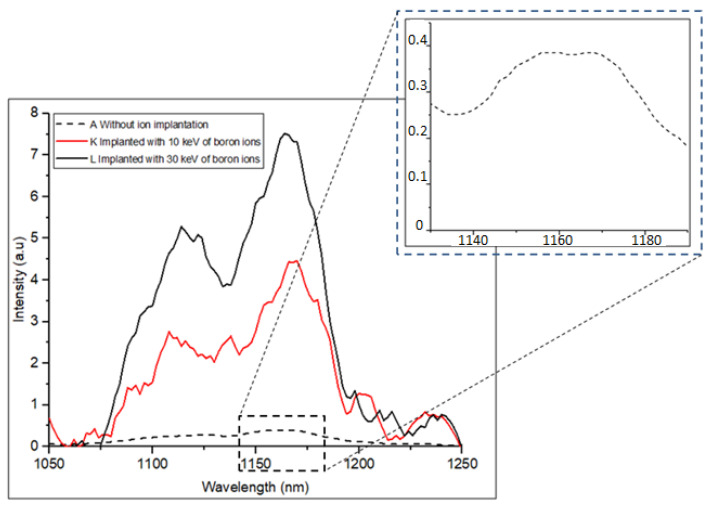
PL spectrum of samples A, K and L at 80 K. Sample A is the reference sample without any implantation. Samples K and L are implanted with 10 and 30 keV of boron ions. The inset shows the peak at 1170 nm for sample A. Laser power is 200 mW. FWHM for sample L is 46 nm (43 meV).

Figure 6 shows the PL emission intensity at 80 K for samples K and L which have been implanted with boron at 10 and 30 keV, respectively. Sample A remains the reference sample without any dopants. Implantation conditions, such as the implant energy and dose, were carefully selected to form the dislocation loops in the lattice, thereby improving the emissions in silicon [23]. As expected, the introduction of boron atoms increased the probability of a radiative recombination route in the silicon structure and improved the emissions of samples K and L compared with those of the intrinsic sample A. The highest peak intensity is observed in sample L, with an improved peak emission of up to 130 times higher than that in reference sample A. The enhancement in emission intensity can also be attributed to the presence of a high concentration of boron in the lattice, leading to a much more effective ion diffusion [25].

**Figure 7:**
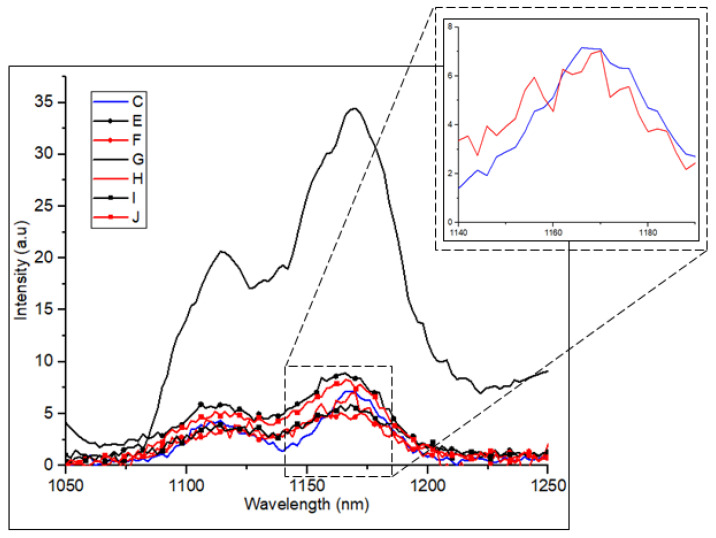
PL spectrum of samples C, E, F, G, H, I and J at 80 K. The black lines represent samples implanted with carbon prior to boron implantation at 10 keV. The red lines represent samples implanted with carbon prior to boron implantation at 30 keV. The blue line is sample C from the previous result, selected as reference. The highest intensity is observed in sample G with 30/10 keV of carbon and 10 keV of boron implantation. The inset shows the peak at 1170 nm for samples C and H. Laser power is 200 mW. FWHM for sample G is 51 nm (49 meV).

A further investigation on the silicon emissions was performed with various samples implanted with carbon and followed with boron at different implantation energies (refer to Table 1). Figure 7 shows the PL emission intensity measured at 80 K with very close peak values at the emission regions of 1050 and 1250 nm for different samples, including C, E, F, G, H, I and J. Sample C, which previously has the highest peak intensity in the samples doped with only carbon, was chosen as the reference sample. The maximum PL peak is observed from sample G, which reflects the enhancement due to the incorporation of the double implantation of carbon ions with energy of 30 and 10 keV (similar to sample C) and 10 keV of boron ions into the silicon substrates. Compared with the other samples, this sample exhibits a seven-fold increase in the bandgap peak emissions. This finding confirms the crucial role of boron and carbon in assisting the enhancement of silicon emissions by forming the radiative damage centers or dislocation loops near the surface of silicon. Despite the same carbon implant condition in samples G and H, the latter, with a higher boron implant energy at 30 keV than the former, exhibits decreased PL emissions comparable with those of the other samples. The high energy of boron in sample H resulted in the dislocation loop formation located further in the substrate and a lessened light absorption efficiency whilst competing with the interstitials to form the dislocation loops. The overall enhancement of the peak intensity in sample G is ~600 times greater than that in the silicon intrinsic sample.

**Figure 8:**
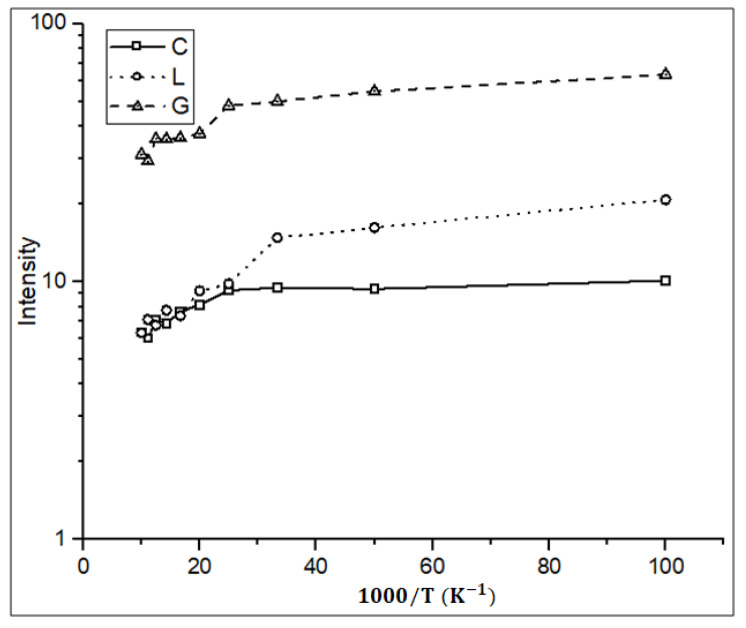
PL intensity of sample C, L and G at 1170 nm against 1000/T. Samples C and G are double implanted with 30/10 keV of carbon without boron implantation and with 10 keV of boron implant, respectively. Sample L is only implanted with 30 keV of boron. Laser power is 200 mW.

Figure 8 shows the temperature dependence of the PL peak intensity at 1170 nm for samples C, L and G. A previous paper has reported the temperature quenching problem using a similar defect-engineered technique [15]. The gradual decline in intensity with the increasing temperature for all the samples shows the classic effect of dislocation engineering being incorporated into the samples [21]. The introduction of dislocation loops improves carrier confinement, thereby eliminating or reducing the nonradiative recombination route whilst simultaneously increasing the radiative recombination. Sample C, doped with only carbon, also shows a similar effect because of the dislocation loop formation by carbon specific implantation energy and annealing temperature.

**Figure 9:**
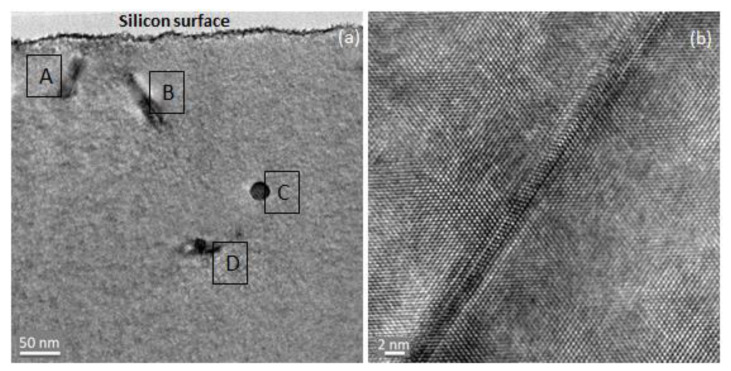
TEM analysis for (**a**) sample G implanted with 30/10 keV of carbon and 10 keV of boron ions and (**b**) for point A. The defects A and B can be clearly seen at the depths of 100 nm and 200 nm. The defects C and D are the deep-level trap.

The low-magnification HRTEM image in Figure 9a confirms the existence of the dislocation loops. Dislocations are one-dimensional crystal defects in which their properties depend on the crystal symmetry. The dislocation loops labelled A and B can be clearly seen depths of around 100 nm and 200 nm. The defects labelled C and D are deep-level traps which will not influence further nano- or micro-scaled device fabrication in silicon. A magnified HRTEM image of one of the loops is shown in Figure 9b. The dislocation in the TEM image is characterized by distortions of the periodic lattice structure around the defects, as shown in Figure 9b. The TEM analysis shows that the controlled implantation of carbon and boron in a silicon lattice structure will result in producing sufficient number of interstitials which subsequently form the dislocation loops. The presence of dislocation loops causes a difference in pressure at the interface of the loop and the neighboring silicon lattice. Since bandgap energies in semiconductors are pressure-dependent, the bandgap energy around the loop is increased by ~0.75 eV and creates a potential barrier that prevents the carrier from conventionally recombining non-radiatively [21]. In the absence of the non-radiative recombination route, the carriers need to recombine via the radiative route, thus improving the efficiency of light generation in silicon.

**Figure 10:**
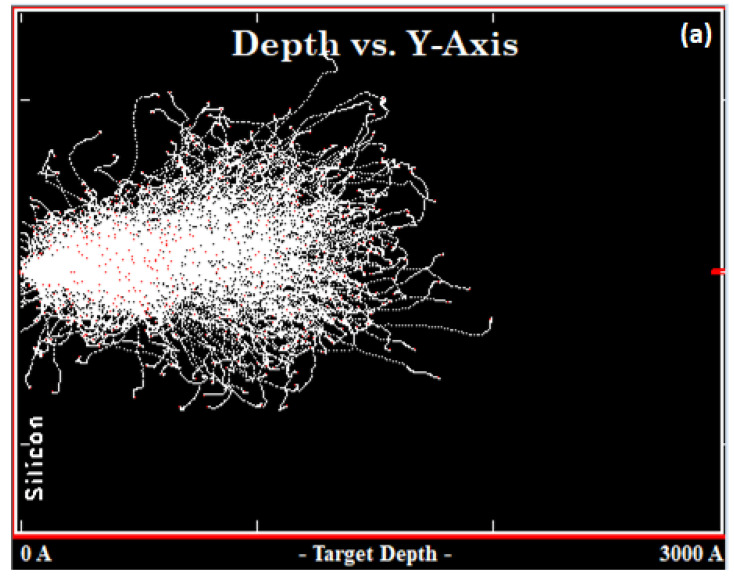

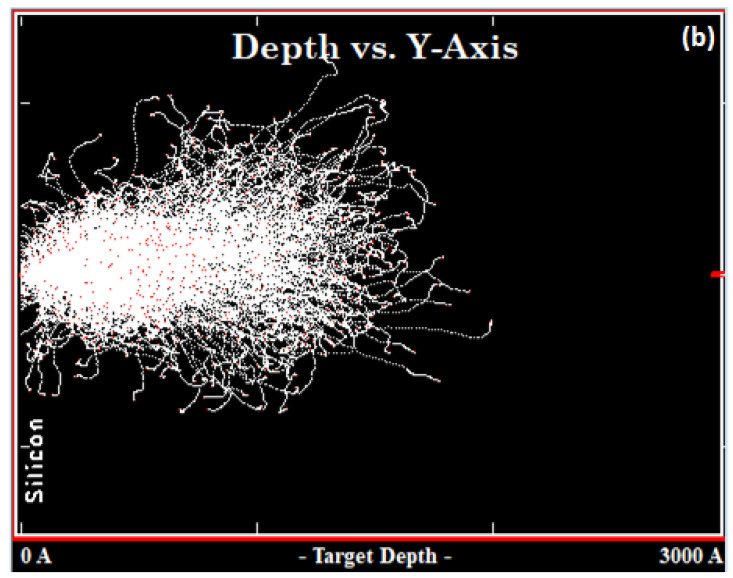
SRIM result for samples (**a**) C and (**b**) G. The ions’ distribution in the silicon sample can be seen in both implant profiles.

The Stopping and Range of Ions in Matter (SRIM) simulation, which is based on the Monte Carlo implant model, is used to study the behavior and interactions of ions with matter [26]. A SRIM simulation analysis was performed for samples C and G to observe the effect of carbon and boron implantation on the defect production and distribution. Figure 10 shows the SRIM results of the ion distribution after carbon implantation in sample C, as well as boron and carbon implantation in sample G.

The difference between these two samples is the distribution of the ions near the surface of silicon. The ion displacements after implantation, which improve the radiative recombination, are more evenly spread near the surface of the silicon in sample G than in sample C. The depth of the dislocation loops is predicted by using the SRIM calculation by assuming that the loops reside within the depth of boron and carbon implantation. This scenario resulted in an emission intensity in sample G that is higher than that in sample C, as shown in Figure 7, in which the implanted ions penetrate a depth up to ~250 nm (2500 Å) and spread up to ~100 nm (1000 Å) wide. This result agrees with the finding in HRTEM (Figure 9) which gave the exact location of the loops.

## 4. Conclusions

In conclusion, we have successfully designed a new technique to efficiently extract light silicon by the double implantation of carbon, followed by boron, with a specific implantation energy. After each implantation, the samples were annealed to assist in the creation of dislocation loops whilst activating dopant mobilization. PL measurements were performed to observe the emission peaks and their intensities. The highest emission peak was observed in sample G with an increment of ~600 times compared with the same peak in the intrinsic silicon sample. An improvement in the temperature quenching issue was also observed, proving the presence of dislocation loops in the lattice structure. The existence of the dislocation loops has also been proven. These promising results are crucial in the development and realization of all silicon-based photonic systems, such as optical communication, sensing and any other related products.

## Figures and Tables

**Table 1:** Details of the samples.

Sample	Carbon Implantation				Boron Implantation	
	1st Implant		2nd Implant			
	Energy (keV)	Dose (Ion cm^−2^)	Energy (keV)	Dose (Ion cm^−2^)	Energy (keV)	Dose (Ion cm^−2^)
A	-	-	-	-	-	-
B	30	4.0 × 10^13^	-	-	-	-
C	30	4.0 × 10^13^	10	1.1 × 10^13^	-	-
D	50	4.0 × 10^13^	20	1.1 × 10^13^	-	-
E	30	4.0 × 10^13^	-	-	10	1.0 × 10^15^
F	30	4.0 × 10^13^	-	-	30	1.0 × 10^15^
G	30	4.0 × 10^13^	10	1.1 × 10^13^	10	1.0 × 10^15^
H	30	4.0 × 10^13^	10	1.1 × 10^13^	30	1.0 × 10^15^
I	50	4.0 × 10^13^	20	1.1 × 10^13^	10	1.0 × 10^15^
J	50	4.0 × 10^13^	20	1.1 × 10^13^	30	1.0 × 10^15^
K	-	-	-	-	10	1.0 × 10^15^
L	-	-	-	-	30	1.0 × 10^15^

Sample A is the reference sample without any ion implantation. B, C and D are samples without boron implantation. Sample E, F, G, H, I and J are implanted with carbon and boron ions. Samples K and L are sample implanted with boron ions only.

## Data Availability

Data available on request due to restrictions, e.g., privacy or ethical. The data presented in this study are available on request from the corresponding author.
